# Forensic DNA databases in Western Balkan region: retrospectives, perspectives, and initiatives

**DOI:** 10.3325/cmj.2011.52.235

**Published:** 2011-06

**Authors:** Damir Marjanović, Rijad Konjhodžić, Sara Sanela Butorac, Katja Drobnič, Siniša Merkaš, Gordan Lauc, Damir Primorac, Šimun Anđelinović, Mladen Milosavljević, Željko Karan, Stojko Vidović, Oliver Stojković, Bojana Panić, Anđelka Vučetić Dragović, Sandra Kovačević, Zlatko Jakovski, Chris Asplen, Dragan Primorac

**Affiliations:** 1 Institute for Genetic Engineering and Biotechnology, Sarajevo, Bosnia and Herzegovina; 2 Genos doo, Zagreb, Croatia; 3 Clinical Center University of Sarajevo, Sarajevo, Bosnia and Herzegovina; 4 Ministry of Science, Education, and Sports, Zagreb, Croatia; 5 Faculty of Criminal Justice and Security, University of Maribor, Maribor, Slovenia; 6 National Forensic Laboratory, Police, Ministry of the Interior, Ljubljana, Slovenia; 7 Forensic Science Center “Ivan Vučetić,” Ministry of Interior, Zagreb, Croatia; 8 University Center for Forensic Sciences, University of Split, Split, Croatia; 9 Ministry of Interior Federation of Bosnia and Herzegovina, Sarajevo, Bosnia and Herzegovina; 10 Center of Legal Medicine of Republic of Srpska, Banja Luka, Bosnia and Herzegovina; 11 Faculty of Medicine, University of Banja Luka, Banja Luka, Bosnia and Herzegovina; 12 Institute for Legal Medicine, Belgrade, Serbia; 13 DNK Centre for Genetics, Belgrade, Serbia; 14 National Crime-Technical Centre, Ministry of the Interior, Belgrade, Serbia; 15 Forensic Center, Police Directorate, Danilovgrad, Montenegro; 16 Institute of forensic medicine, criminology and medical ethics, School of Medicine, University “St. Cyril and Method,” Skopje, Republic of Macedonia; 17 Gordon Thomas Honeywell Governmental Affairs, Washington DC, USA; 18 Medical School, University of Split, Split, Croatia; 19 Eberly College of Science, Penn State University, University Park, Pa, USA; 20 University of New Haven, New Haven, Conn, USA

## Abstract

The European Network of Forensic Science Institutes (ENFSI) recommended the establishment of forensic DNA databases and specific implementation and management legislations for all EU/ENFSI members. Therefore, forensic institutions from Bosnia and Herzegovina, Serbia, Montenegro, and Macedonia launched a wide set of activities to support these recommendations. To assess the current state, a regional expert team completed detailed screening and investigation of the existing forensic DNA data repositories and associated legislation in these countries. The scope also included relevant concurrent projects and a wide spectrum of different activities in relation to forensics DNA use. The state of forensic DNA analysis was also determined in the neighboring Slovenia and Croatia, which already have functional national DNA databases. There is a need for a ‘regional supplement’ to the current documentation and standards pertaining to forensic application of DNA databases, which should include regional-specific preliminary aims and recommendations.

Different methods of forensic DNA testing (known as DNA typing) have been widely used as standard procedures in police investigations and court testimonies. In the last 15 years, the primary value of these procedures has been considerably increased by the introduction of polymerase chain reaction and analysis of short tandem repeat (STR) loci. STR loci have been proven to be a highly reliable and powerful tool in paternity testing, as well as in forensic and mass disaster human identification (1-8).

The success of DNA testing in a police investigation can be greatly enhanced by storing DNA profiles in a central database. Alternatively, DNA testing can only be useful once police investigators have identified a suspect by traditional investigative methods. However, with the DNA database, the investigative process becomes more efficient, especially in cases where traditional investigative techniques have been unhelpful and no suspect can be identified (9,10). Since they are aware of their great investigative potential, more and more countries and law enforcement agencies are creating and utilizing DNA databases.

In the Western Balkan region, the primary value of DNA analysis in forensic sciences has significantly increased since the early 1990s when identification of human remains from hundreds of mass graves began. These missions in Bosnia and Herzegovina (11,12), Croatia (13), Macedonia (14), and Kosovo (11) identified and solved a huge number of completely unforeseen difficulties in the forensic DNA analysis of skeletal remains and databasing of the obtained results.

Through the experience and knowledge gained through the identification of war victims, a reputable and strong scientific community in the field of forensic genetics has been established. Currently, more than 20 functional forensic DNA laboratories are operational in this region. They have also been involved in other new challenges, such as the DNA identification of skeletal remains from the World War II mass graves (15,16).

However, the legislative and DNA database developments in most countries of the Western Balkan region have not followed DNA technology developments, although it has been recognized that efficient national forensic DNA databases and a high level of expert networking should be one of the priorities for the local and national forensic scientific community. Therefore, in March 2011 the first regional workshop on this topic took place in Sarajevo, Bosnia and Herzegovina. Scientists from the Western Balkan region decided that the very first step in development of sustainable forensic DNA databasing strategy would be the documentation of our knowledge about the existing forensic DNA data repositories and associated legislation in Bosnia and Herzegovina, Serbia, Montenegro, and Macedonia, as well as in the neighboring Slovenia and Croatia, which already have functional DNA databases. The investigation also included relevant concurrent projects and a wide spectrum of different activities in relation to forensics DNA use in these countries. This article is the first result of our joint efforts in this domain (Figure 1).

**Figure 1:**
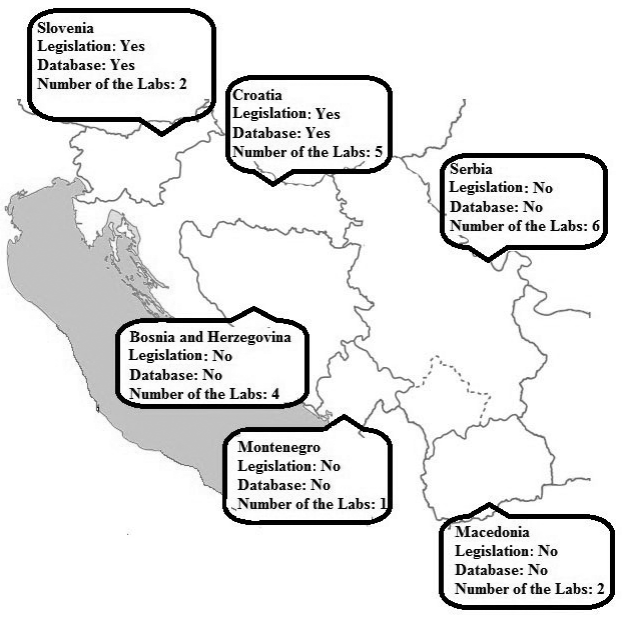
Overview of the legislation, databases, and the number of the laboratories in Slovenia, Croatia, Bosnia and Herzegovina, Serbia, Montenegro, and Macedonia.

## DNA database in Slovenia

The Slovenian forensic DNA database was legally established in 1998 (17), thus making Slovenia the first country in the Western Balkan region with a national forensic DNA database. The criteria for DNA profiles inclusions in the database are determined by the Police Act. According to the profile entry criteria, all suspects’ and crime scene evidence profiles can be submitted. Suspects’ profiles are removed from the database upon the acquittal and crime scene evidence profiles are removed when a match occurs. The storage of convicted offenders’ profiles varies according to the type of crime. There is no specific legislative provision for retention or destruction of the physical samples. In practice, physical samples from individuals are destroyed after a DNA profile has been obtained. After the analysis, crime scene-related samples are returned to the police officer in charge or a magistrate.

In addition to the Police Act, the Criminal Procedure Act (18) was amended to explicitly allow the police to, besides fingerprinting and photographing, take buccal swabs from suspects. With such legislation, the DNA database is placed at the same procedural level with other police records, especially the fingerprints records.

The Slovenian Police uses an internally developed DNA database software that recognizes two categories of DNA profiles: persons and crime scene evidence. Between 1998 and 2001, the DNA database was located at the DNA laboratory of the National Forensic Laboratory (NFL) and was not directly accessible for other users in the police. Since 2001, the database can be accessed online, by authorized personnel only via the police Intranet. Each forensic department within regional police directorates is obligated to enter all legally prescribed data on the samples submitted for DNA profiling contained in the database. Authorized DNA laboratory experts then provide DNA profiles and other required data for these entries. Only the director of the forensic DNA laboratory is authorized to delete or make significant changes of the records.

The DNA database can be cross-referenced to the other police records. These interfaces guarantee that the individual DNA profiles are automatically deleted if they refer to individuals who have been acquitted or served their sentences, or if there are other legal reasons for deletion (19).

Prior to 2007, international data exchange was performed via the Slovenian national bureau located in Ljubljana. In 2007, Slovenia ratified the Prüm treaty (9), which permits international DNA information exchange. Since then, the DNA data are exchanged on a daily basis directly with the other Prüm signatory states. This exchange encouraged the expansion of the number of STR loci used for the DNA analyses. Currently, the DNA profiles loaded in DNA database are analyzed for 15 STR loci, including all from the expanded European Standard Set.

In April 2011, there were almost 19 000 DNA personal and crime scene evidence profiles stored in the Slovenian DNA database. Convicted individuals’ records are not stored separately within the database. With the DNA database growth, the number of cold hits (crime scene evidence to persons and evidence to evidence profile) is also increasing. In 2010, there were approximately 500 person-to-stain and 100 stain-to-stain cold hits. Since the DNA database implementation in Slovenia, more than 3000 cold hits, as defined by the ENFSI DNA working group recommendations, have been recorded. The majority of all recorded hits were related to break-ins and thefts of motor vehicles, followed by robberies and other criminal offenses. Some of the cold hits were used for identification of murder suspects. The numbers of cold hits and strict compliance to the ENFSI recommendations indicate that the Slovenian DNA database and its associated legislation are highly efficient assisting the investigation, but also in respecting the citizens’ privacy.

In addition to the NFL, where almost all forensic DNA analysis in Slovenia is performed, another forensic DNA laboratory is located at the Institute of Legal Medicine, Medical Faculty, Ljubljana University. However, the NFL is the only laboratory that contributes records to the Slovenian DNA database. All the mentioned activities have been followed by the genetic studies of the Slovenian human population and precise determination of all the required statistical parameters used in the presentation of DNA profiling results (20-24).

## DNA database in Croatia

Croatia is one of the first countries in the world where mass graves skeletal remains were successfully identified by the use of DNA analysis (2). Before the identification process commenced, Croatian DNA population studies had been performed to determine all the statistical parameters required in the presentation of DNA profiling results (25-28). Currently, Croatia has 5 laboratories capable of doing forensic DNA analysis: Forensic laboratory at the Ministry of the Interior; Forensic laboratory at the University of Zagreb School of Medicine; Forensic laboratory at the University Hospital Split; Forensic laboratory at the University of Osijek School of Medicine, and a privately owned laboratory “Genos.”

In 2001, the first Croatian forensic DNA database was established at the Forensic laboratory of the Ministry of the Interior located in the Forensic Science Center “Ivan Vučetić.” Combined DNA Index System (CODIS) software has been in official use since 2006, and all DNA profiles are stored in accordance with the international recommendations of ENFSI and INTERPOL. The Croatian DNA database is divided into the following categories: suspect profile, forensic sample profile, forensic mixture profile, staff profile, and others. In April 2011, it contained about 29 500 DNA profiles developed from suspects and about 4000 DNA profiles from disputed traces. Since its establishment, more than 1000 hits have been recorded.

Through the new Criminal Procedure Act in 2008 (29), Croatia extended the number and type of criminal offenses for which DNA forensic analysis of reference and casework samples can be performed. Thus, the provision of an expert opinion can now be ordered whenever it may provide useful information on a criminal offense. However, it is obligatory for criminal offenses with a minimum prison sentence of 6 months, along with the expert opinion before and during the criminal procedure. The retention period for storing and keeping data collected through forensic DNA analysis was increased from 10 years to generally 20 years following the completion of the criminal procedure. The conditions that will have to be met to allow biological material to be stored for even longer than 20 years will be regulated by a bylaw. These conditions should regulate data deletion, taking reference and casework samples, storing, processing, keeping, and storage supervision, and processing and keeping of biological samples and obtained data.

In addition, Enforcement of Prison Sentence Act (30) has been amended to ensure that a biological sample is taken from all prisoners convicted of a criminal offense with the minimum prison sentence of 6 months, and from those who will be serving a prison sentence when the amendments to the Act come into force. This could be used to prevent further criminal offenses by the same person. These amendments of the law should increase the efficiency of the Croatian criminal law system and the Croatian legal system as a whole (31).

## DNA database in Bosnia and Herzegovina

The current forensic DNA practice in Bosnia and Herzegovina (BH) takes place through two different, relatively separate processes. The first is the identification of the war victims by DNA testing, led by the International Commission of Missing Persons (ICMP). Since November 2001, ICMP has developed a database of 88 610 relatives of 29 073 missing people, and more than 33 000 bone samples have been taken from mortal remains exhumed from clandestine graves in the countries of former Yugoslavia. By matching DNA from blood and bone samples, ICMP has identified 15 955 people who went missing during the conflicts and whose mortal remains were found in hidden graves (data downloaded from *www.ic-mp.org*). So far, this assignment has produced very important evidence for the war-crime trials conducted at the UN War Crimes Tribunal at The Hague. It also aims to provide answers to the thousands of BH citizens who are still looking for the remains of their beloved ones to give them a dignified burial (32,33). In the last 10 years, up to 200 000 DNA profiles have been generated and significant scientific information has been collected. Also, BH scientists have identified and resolved a multitude of completely unforeseen difficulties in the DNA analysis of skeletal remains. This has all enabled them to become leading experts in forensic DNA anthropometric genetics who are now helping in the identification of the mass-disaster victims all over the world.

The second process is the DNA analysis for forensic police investigation and routine parentage testing. These two procedures are performed routinely in several laboratories, which are acting under the orders from the district attorneys, courts, lawyers, or at personal requests (paternity testing).

Prior to 2010, forensic DNA laboratories, situated at two scientific institutions (Institute for Genetic Engineering and Biotechnology and Clinical Center University of Sarajevo) carried out the biggest share of the testing load. In the last two years, the Federal Ministry of the Interior has established its own laboratory through an EU-program, and has taken over the majority of crime scene biological evidence analysis. More recently, the Center for the Forensic Medicine of the Republic of Srpska (Banja Luka) has also established its own forensic DNA laboratory. BH now has an extensive experience in the implementation and application of forensic DNA analysis and DNA profile registries. Additionally, the genetic structure of Bosnian population has been comprehensively investigated and documented (34-39).

Unfortunately, the existing BH legislation system is not following the scientific achievements in this field. Whole forensic DNA analysis is covered by only 4 paragraphs of the Law of Criminal Procedure. Due to this lack of legislation, there is a big risk for misinterpretation of the DNA analysis results and potential for its abuse (40). Consequently, DNA evidence could be easily challenged or even rejected in court.

In January 2010, the Ministry of Justice submitted a new Act for Application of DNA Analysis Results in Court Trials to the Parliament. Unfortunately, this document was created without cooperation with the forensic scientific community, ignoring many of the existing practices of DNA testing, its standards and recommendations. In 2010, the Act was stopped at the Parliament, but even if it is passed, it is not going to result in any practical improvement in the application of forensic DNA analysis in BH. Hopefully, the joint efforts of the Governmental Agency for Forensics (in charge of the BH DNA database development) and the BH forensic DNA community will help to make improvements in the field.

## DNA database in Serbia

Forensic DNA testing in Serbia is performed in 6 laboratories, most of which participate in the GEDNAP proficiency testing program (41). Forensic DNA analysis has been conducted in Serbia since 1997, when the use of the polymarker TM system at the Faculty of Biology, the University of Belgrade began. Nevertheless, a major technological breakthrough was the installation of the ICMP facility at the Institute of Legal medicine in Belgrade in 2002, in which the state of the art fluorescent chemistry-based DNA typing technology was used to type 18 autosomal loci. Also, detailed genetic studies of the Serbian human population, using these loci, has been performed during the last few years (42-46).

The use of DNA typing in criminal procedures in Serbia is regulated by the Criminal Procedure Act, which empowers the district attorney to order the police to collect the buccal swab samples from the suspect, with or without his or her consent. The same law requires the medical examiner to collect the DNA samples from unidentified bodies, and to collect and preserve samples of any biological traces (blood, urine, saliva, hair, semen etc.) obtained during autopsy. The Law on the Police allows the police to make a registry of individuals subjected to DNA testing during criminal investigations, although there is no specific mention of how DNA profiling results are maintained. As a result, any unidentified DNA profile developed in one laboratory during the criminal investigation is sent by the investigative judge to all other laboratories for comparison with internal DNA registries, which is a time-consuming process. In order to further support the establishment of DNA databases, a working group was formed in 2004 by the Ministry of Justice, which drafted the Law on a National DNA Registry. While awaiting the enactment of the law, the Section of Forensic Medicine of the Serbian medical society has issued several guidelines on the use of DNA testing, mainly for paternity cases and DNA-assisted identifications of dead persons.

## DNA database in Montenegro

Taking samples for DNA analyses by the Police in Montenegro is regulated by the Criminal Procedure Law (47), which empowers police authorities to obtain a sample from persons and collect evidence for DNA analysis. DNA profiles obtained from suspect reference samples and DNA profiles obtained from evidence material of unresolved criminal cases are incorporated into the DNA database of the Forensic Center. The police forensic DNA laboratory started working independently in August 2009, and it is the only laboratory in Montenegro that performs forensic DNA analyses. DNA analyses are carried out based on the request of the police, court, or state prosecutor.

DNA profiles are stored pursuant to the Law on Police (48), which permits keeping personal records, fingerprints, photographs, and DNA profiles for the identification purposes. Profiles from a database are regularly checked, which has resulted in a dozen cold hits. In April 2011, the database contained around 2000 reference profiles. The new Criminal Procedure Law (49) enacted in 2009, regulates taking of samples or biological material for DNA analyses, packaging, storage, and processing of biological material, and storage of DNA samples and the results of DNA analyses, which must be performed pursuant to the individual law.

On February 17 2011, the Government of Montenegro accepted the action plan (50) following all general recommendations of the European Commission. One of the goals is to recommend a draft of legislation on the national DNA registry until the end of June 2011, which should regulate forensic DNA databasing issues that are still open.

## DNA database in the Republic of Macedonia

The legislation of the Republic of Macedonia regulates the process of performing DNA analysis for the purpose of establishing facts in criminal and legal procedures. Although, according to Criminal Procedure Law (51), the court cannot explicitly allow conducting such analysis during the procedure, it is still one of the most important investigating instruments in the criminal procedure in compliance with the regulations of “testifying in court as an expert.” The Law on Criminal Procedure regulates the conditions for physical examinations of the accused, including performing venipuncture to obtain blood sample. Any medical examination must be performed according to the rules of medical science and may only be performed to establish findings relevant for the criminal procedure, without taking risk to impair the health. It is not allowed to perform medical interventions on the accused or the witness or to give them medicaments that would influence their will when making the statement.

In November 2010, the Parliament of the Republic of Macedonia passed the Criminal Procedure Law (52) as part of harmonization of the Republic of Macedonia criminal legislation with the European legislation. This law has finally accepted DNA analysis results as evidence in criminal investigation procedures. Also, it regulates taking of fingerprints, samples for DNA analysis, and photographs.

The Republic of Macedonia legislation is in the process of harmonization with the European Law on police cooperation. In that context, the Chapter 24 “Justice, Freedom and Security of the National Program for Adoption of the Acquis Communautaire 2011” has anticipated the adoption of legislation on standardization of the DNA procedures. The following EU regulations will be taken into consideration: Council Resolution of June 9, 1997 on exchange of DNA analysis results (97/C 193/02) and the Council Resolution of June 25, 2001 on exchange of DNA analysis results (2001/C 187/01)”.

To increase efficiency in international legal cooperation regarding the fight against organized crime, Republic of Macedonia legislation should provide competent authority for record keeping, collecting and processing DNA data, data protection, international data exchange, time period for storing biological evidence, DNA samples from minors, etc.

There are two Forensic DNA laboratories in the Republic of Macedonia. One is a part of the Institute of Forensic Medicine, Criminology and Medical Deontology and is an independent scientific institution protected by the autonomy of the “St. Cyril and Method” University. The other is the forensic laboratory associated with the Ministry of the Internal Affairs of the Republic of Macedonia. These laboratories assisted in performing comprehensive population genetic studies of the Macedonian population (53-57).

## Forensic databases in Europe and the USA

Now that unmonitored border-crossing in the European Union is a reality, people can move freely between countries without being questioned or checked against any databases. This applies not only to law abiding citizens but also to criminals. For example, if a paroled pedophile just crosses the border, the potential of his DNA profile stored in his home country would be rendered useless (9).

Therefore, in 1997 the Council of the European Union invited Member States to consider establishing DNA databases (58). In 2009, previously established European Standard Set (ESS) was expanded up to 12 loci (59). Since 2008, DNA-working group of the ENFSI has been responsible for making updated DNA database reviews and recommendations (60). Currently, this document summarizes 28 different recommendations regarding legislation, matching criteria, accreditation issues, removal procedures, automatization of the database procedures, and several other topics.

In the United States, DNA databases are designed around the CODIS software system. The CODIS databases exist at the local, state, and national levels. This tiered architecture allows crime laboratories to manage the data from their own jurisdictions as well to share their data. Each state decides which profiles will be included in the state database. These profiles are then shared nationally according to Federal Bureau of Investigation (FBI) policy and Federal law. As of 2011, approximately 180 laboratories in all 50 states participate in the CODIS. The national connectivity between all 50 States is managed by the National DNA Index System (NDIS), operated by the FBI. In April 2011, the database contained 361 176 forensic profiles and 9 404 747 offender profiles, making it the largest DNA database in the world. CODIS has produced over 138 700 matches to requests, assisting in more than 133 400 requests.

The FBI takes an active role in assuring the quality of the results in the database. The DNA Advisory Board was established to develop and revise the recommended standards for quality assurance. It fulfilled its mission by recommending two quality assurance documents to the Director of the FBI, resulting in the issuance of the Standards for Forensic DNA Testing Laboratories and Standards for Convicted Offender Laboratories (61).

## Recommendations

The single most important factor contributing to an effective DNA database is the legislation describing the creation of the database and its legal use. Even when there is excellent laboratory analysis and short processing time, ineffective database legislation can reduce the potential of DNA databases. It is the quality of DNA database laws that make DNA an effective investigative tool.

Different countries have developed different approaches to implementation and use of DNA technology. Issues that must be addressed in the development of forensic databases include the type of records included in the database, storage of physical samples, length of profile retention, and expunging of records (9). Given the current situation in some of the Western Balkan countries, all activities regarding forensic databases legislation should be done in cooperation with local forensic DNA scientific experts. The existing official EU recommendations (58-60) as well as the US, Slovenian, and Croatian experiences could be very beneficial for achieving this goal.

*Recommendation #1:* Regional state authorities in Bosnia and Herzegovina, Serbia, Montenegro, and Macedonia should establish efficient, functional, and progressive national legislation for the creation and usage of DNA databases.

A functional DNA database is an exceptionally potent tool in crime solving and prevention, and in the protection of the innocent. However, like any other sophisticated tool, it requires very specific operational conditions. If mishandled, the database may transform from a problem solving tool to a problem-causing mechanism. Once an STR profile is downloaded into a database, it will be continuously compared with both the reference and crime scene profiles over a long period of time. If a profile containing an error was recorded in the database, it would be nearly impossible to identify it in the later stages. Therefore, it is paramount to implement legal mechanisms that will minimize errors, with high level of certainty for the DNA process.

*Recommendation #2:* Regional state authorities in Bosnia, Serbia, Montenegro, and Macedonia should establish operative DNA databases according to the existing EU criteria, while respecting their own specific DNA testing practices.

There is an increased potential to share data among countries, especially when more countries establish and expand their forensic DNA databases. Some countries without specific DNA legislation have already started the establishment of DNA databases under other criminal justice authorities. As these databases increase in size and effectiveness, the value of sharing data across borders becomes apparent (9). The Prüm Treaty (9), which was signed by 11 countries, facilitates the exchange of DNA data between all signatories. Recently, EU legislation has made all 27 members of the European Union to participate in a network of national DNA databases. Given this legislative development, criminals will no longer be able to avoid law enforcement’s most effective crime-fighting tool by simply crossing the border (9). This is especially important in this part of Europe, which until recently belonged to one country and in some places the borders are still not precisely determined, which allows criminals to move freely between countries.

*Recommendation #3:* Regional state authorities should provide efficient and continuous data sharing among the existing and future DNA databases in the Western Balkan region, as well with other countries.

As documented above, countries of the ex-Yugoslavia region have a long and distinguished tradition of using forensic DNA technology, especially in the development of databases and application of new technologies for the analysis of low-template DNA samples. Also, regional scientists have already conducted many studies on the genetic characteristics of the population. This, together with the experience from other countries, provides not only a solid foundation for the development of the databases, but also allows the choice of a suitable STR set of loci (respecting the previous ENFSI recommendations) and a proper interpretation of DNA profiles produced from low-template DNA sample.

*Recommendation #4:* Regional state authorities should employ the existing regional forensic experience in the development of suitable DNA databases.

*Recommendation #5:* Chosen set of STR loci should meet the standards that will allow data-sharing with other EU databases, but with respect to the genetic specificity of the local population.

*Recommendation #6:* Existing regional forensic experience in application of new technologies for the analysis of DNA profiles produced from low-template DNA samples should be used in development of a matching strategy for this kind of sample.

Previous regional experience with the DNA databases shows that the majority of problems with database entries have been caused by manual typing errors and a lack of reference documentation for the profiles. The importance of data quality assurance prior to the database entry cannot be overstated, so it is paramount that regional experts take part in this process.

*Recommendation #7:* It is highly recommended to create Standard Operational Procedures to handle detailed data entry.

Although almost every state has installed its own entry and removal criteria, in the EU there are theoretically 3 different systems that could be applied: a database that contains DNA profiles of all citizens; a system that stores DNA profiles only of those individuals who are suspected or convicted of committing a particular type of crime; or a system that *sensu stricto* does not involve a database – DNA profiles are only created for individuals who are suspected of being involved in a particular criminal offense and are only used in the criminal investigation concerned (62).

Although the first system is currently not in use anywhere in the world, it has been suggested that the creation of population-wide forensic DNA databases could prove to be the most successful crime-fighting measure in history (62). However, a complete forensic DNA database will possibly lead to the violation of a number of individual and civil rights. This is a very sensitive ethical issue, especially in the countries with existing huge DNA human identification databases (Bosnia and Herzegovina, Croatia etc.)

*Recommendation #8:* DNA profiles and other personal data gathered for DNA databases, which are established for the specific purpose of the identification of mass disaster victims, should be protected and not used as a “source of information” for forensic criminal investigation.

These 8 recommendations could form a specific “regional supplement” to the current documentation and standards, which would support and inform the establishment of efficient forensic DNA databases in this part of Europe.

Finally, the DNA forensic science has come a long way in a relatively short period of time. It is very important that both the legislative and database development is done in such a way that keeps them flexible for an introduction of new testing techniques or an addition to the existing ones. There is a thin line between a clear regulation, which ensures a proper functioning, and overregulation. Crossing this line carries a considerable risk of making the newly formed databases obsolete in the near future.
